# Bio-Signal Complexity Analysis in Epileptic Seizure Monitoring: A Topic Review

**DOI:** 10.3390/s18061720

**Published:** 2018-05-26

**Authors:** Zhenning Mei, Xian Zhao, Hongyu Chen, Wei Chen

**Affiliations:** 1 Center for Intelligent Medical Electronics (CIME), School of Information Science and Engineering, Fudan University, Shanghai 200433, China; znmei16@fudan.edu.cn (Z.M.); xzhao17@fudan.edu.cn (X.Z.); 2 Department of Industrial Design, Eindhoven University of Technology, PO Box 513, 5600 MB Eindhoven, The Netherlands; Hongyu.Chen@tue.nl; 3 Shanghai Key Laboratory of Medical Imaging Computing and Computer Assisted Intervention, Shanghai 200032, China

**Keywords:** epileptic seizure, non-stationary signal processing, nonlinear dynamics, complex network, machine learning

## Abstract

Complexity science has provided new perspectives and opportunities for understanding a variety of complex natural or social phenomena, including brain dysfunctions like epilepsy. By delving into the complexity in electrophysiological signals and neuroimaging, new insights have emerged. These discoveries have revealed that complexity is a fundamental aspect of physiological processes. The inherent nonlinearity and non-stationarity of physiological processes limits the methods based on simpler underlying assumptions to point out the pathway to a more comprehensive understanding of their behavior and relation with certain diseases. The perspective of complexity may benefit both the research and clinical practice through providing novel data analytics tools devoted for the understanding of and the intervention about epilepsies. This review aims to provide a sketchy overview of the methods derived from different disciplines lucubrating to the complexity of bio-signals in the field of epilepsy monitoring. Although the complexity of bio-signals is still not fully understood, bundles of new insights have been already obtained. Despite the promising results about epileptic seizure detection and prediction through offline analysis, we are still lacking robust, tried-and-true real-time applications. Multidisciplinary collaborations and more high-quality data accessible to the whole community are needed for reproducible research and the development of such applications.

## 1. Introduction

Epilepsy is one of the most common neurological dysfunctions, affecting about 1% of the world population [1]. Recurrent epileptic seizures accompanied with their neurobiological and cognitive consequences impact patients with epilepsy negatively and consistently. As a chronic disease with age-related features, epilepsy has a huge adverse effect on the quality of life (QoL) of patients, including cognitive impairment, decreased ability of daily activities and the possible social stigma. To date, a fully understanding of the etiology of epilepsy is not yet available. The onset of an epileptic seizure is usually accompanied by electrophysiological anomalies and/or behavioral manifestations. The abnormal synchronized discharge of groups of neurons can happen to the whole brain or originates from several foci and propagates to the whole hemisphere and even the contralateral hemisphere. The clinical manifestations of seizures vary from uncontrolled convulsions of limbs to impaired awareness and pain. Anti-epilepsy drugs are commonly used in the treatment of epilepsy, but it is reported that there are about 25% of patients with epilepsy have drug-resistant epilepsy [2]. Long term freedom from seizures was observed in patients with drug-resistant epilepsy after having the epileptogenic zone resected by surgery with the help of neuroimaging techniques and intracranial EEG to identify the location of it. However, for temporal lobe epilepsy (TLE), one-third of the patients still suffer from recurrent seizures and other complications after surgery. This may be due to poor localization of the epileptogenic zone.

Observations and recordation on epilepsy date back to almost 2000 years B.C [3]. The first international scheme on the classification of epileptic seizures was proposed by the International League Against Epilepsy (ILAE) in 1964 [4]. Epileptic seizures are classified into one of the five main groups by different electroencephalographic expressions during ictal and inter-ictal period, wherein the anatomical and etiological aspects of different seizure types are also discussed. 

In 1969, a revised version [5] of the classification of epileptic seizures proposed in 1964 was given to reflect the new knowledge and the contradictions between experts in this field. The term ‘ictal electroencephalographic expression’ used in the scheme of classification in 1964 was replaced by ‘electroencephalographic seizure type’, emphasizing the equal importance of clinical and electroencephalographic manifestations in characterizing different types of seizure. Subtypes of seizures are defined in more detail by clinical and electroencephalographic manifestations, anatomical and etiologic factors and onset age. At the same time, an international classification of the epilepsies [6] was published. Primary generalized epilepsies, secondary generalized epilepsies and undetermined generalized epilepsies are suggested for diagnostic use with their clinical and ictal and inter-ictal electroencephalographic criteria. Since then, the terminologies of epilepsies and epileptic seizures in these documents had been adopted by numerous neurologists and clinicians worldwide. 

In 1981, another revision about the classification of epileptic seizures [7] was launched by ILAE. Different from the two previous schemes, anatomical substrate, etiology and age factors were no longer retained, and epileptic seizures are differentiated via clinical and electroencephalographic (ictal and inter-ictal) manifestations in parallel.

To supplement the former classification schemes whereby a strong emphasis is placed on the separation of individual seizure types, and to refine the use of ‘epilepsies’ as an insufficiently rigorous implication of ‘diseases’, (International Classification on Epilepsies and Epileptic Syndromes) was presented in 1985 [8] with a revised version followed in 1989 [9]. The profound contribution of these proposals is the terminology used for the classification of ‘epileptic syndrome’. Epileptic syndrome is ‘an epileptic disorder characterized by a cluster of signs and symptoms customarily occurring together’ [9]. Different from a ‘disease’, a ‘syndrome’ could be determined without any confirmed etiology or prognosis. Since only few ‘diseases’ are established until then, ‘epileptic syndrome’ maybe a better choice for denoting the diagnostic entity in clinical practice. The epilepsies are divided into ‘generalized epilepsies and syndromes’, ‘localization-related partial or focal epilepsies and syndromes’, ‘underdetermined’ or ‘special syndromes’. For each group, syndromes are categorized by more detailed descriptions on etiology, seizure type and onset age. The terms ‘idiopathic’ and ‘cryptogenic’ are used, for almost all intentions and purposes, as an alternative of ‘primary’ while circumvent possible misunderstandings; ‘symptomatic’ is used for epilepsies with confirmed pathogenesis.

**Figure 1:**
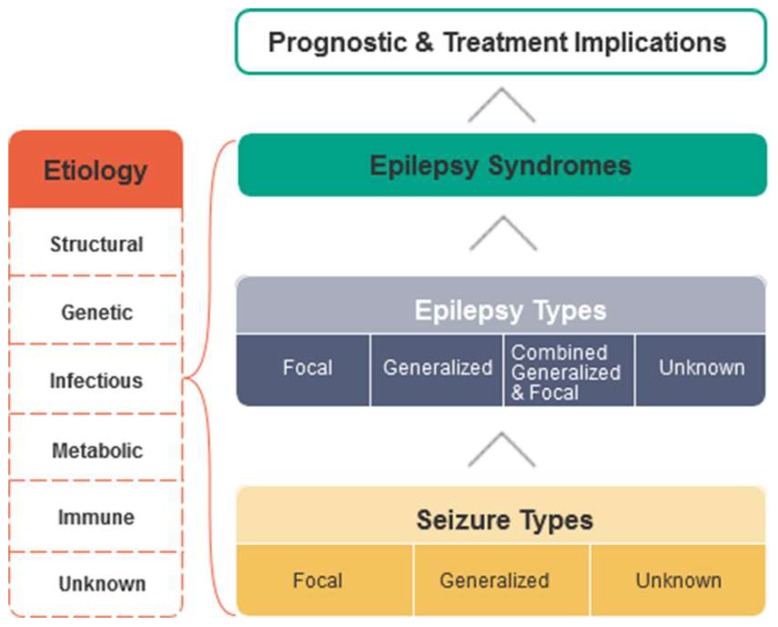
Recommendation of epilepsy and epilepsy syndromes by ILAE.

Currently, the most commonly used definitions of epilepsy and epileptic seizure are those proposed by ILAE in 2005 [10] where an epileptic seizure is ‘a transient occurrence of signs and symptoms due to abnormal excessive or synchronous neuronal activity in the brain’ and a conceptual definition of epilepsy is characterized by ‘enduring predisposition to generate seizures’ and its far-ranging negative effects. It was suggested for the first time that the behavioral disturbances together with the influence from social surroundings caused by recurrent seizures should also be recognized as part of epileptic conditions, to constitute a more comprehensive definition. After that, continuous improvements and modifications [11,12,13] were performed. Figure 1 shows a sketch of the diagnosis of epilepsy or epileptic syndrome recommended by ILAE. Five etiological groups (structural, genetic, infectious, metabolic and immune) are proposed [13] as a shared language to facilitate the communications between neurologists and clinicians with the unknown etiology group intending to cover the patients for whom the cause of epilepsy cannot be identified hitherto.

In clinical practice, the exclusion of non-epileptic seizures together with the classification of epileptic seizures is the first step in the process of the diagnostics and treatment of epilepsy or epileptic syndrome. The identification of the onset and termination of the ictal period is often referred to as epileptic seizure detection or seizure detection. The durations of individual ictal periods, the frequency and intensity of multiple seizures among a specific period and the influence caused by medical interventions can be achieved by retrospective analysis on medical history. Until now, video- electroencephalograms (EEGs) still serve as the gold standard for epileptic seizure detection. They provide both electrophysiological and behavioral information. Epileptologists can inspect the multichannel EEG recordings and the synchronous video recording to identify the indicative electrophysiological and behavioral features from the background activity. During a seizure, the occurrence of visually discernible changes in the EEG and the first clinical manifestation may not happen simultaneously, and the evolution of a seizure which originates from several foci and propagates to a larger field like the entire hemisphere or even both hemispheres can also be reflected by multichannel recordings. For subclinical seizures, behavioral anomalies might not exist and the onset of some specific types of seizures does not have associated EEG reflections. That implies a fully automated seizure detection method robust to all kinds of seizures is not desirable. However, the automatic extraction and labeling of characteristic features of EEG signals is confirmed to be able to accelerate the inspection of EEG recording and identify specific types of seizures. In addition, EEG has good time resolution but fairly limited spatial resolution. On the other hand, well-developed neuroimaging technologies, such as positron emission tomography (PET), single photon emission computed tomography (SPECT), magnetic resonance imaging (MRI), functional magnetic resonance imaging (fMRI) have been widely adopted in the localization of epileptogenic zone, as for the diagnosis of epilepsy [14]. It should be noted that in recent years, several ultrafast neuroimaging techniques have been prototyped and proposed [15,16]. It could be more practical to picture the brain activities without having to make a trade-off between time resolution and spatial resolution. Except for the localization of epileptogenic zone which may cause recurrent seizures, the structural and functional evolution of the brain networks of patients with epilepsy which may be caused by chronic epilepsy can also be reflected by neuroimaging techniques.

For patients with epilepsy, they are more likely to drown because of seizures’ onset during bathing, and Sudden Unexpected Death in Epilepsy (SUEPD) is the biggest epilepsy-related risk factor [1]. In particular, for newborns, epilepsy causes damage to the brain function development, especially for preterm infants. However, most of the patients (about 80%) with epilepsy live in developing countries and do not have access to state-of-art diagnosis, and even in the industrialized country, only a few can get the proper treatment they need. Furthermore, manual inspection the video-EEG of patients places a heavy burden on clinical staff. Long-term continuous video-EEG monitoring prevents the patients from normal daily activities.

For the aforementioned reasons, automatic seizure detection and prediction based on EEG has been approached since the 1970s [17,18]. However, until now long-term EEG monitoring was still limited to highly structured environments, as the potential value of wearable devices capable of continuously and unobtrusively monitoring the related physiological and behavioral signals (ECG, EMG, EDG and motion signal, etc.) was explored [19,20]. For such seizure detection and prediction systems, pattern recognition and machine learning algorithms are adopted to ‘classify’ an epoch of signal into different classes [21]. Some of the classes indicate the onset of seizure or ictal period of a seizure, and an alarm is supposed to be triggered when a specific epoch of signal is classified into such classes, so that the clinician will verify whether a seizure has happened and take necessary medical intervention timely. Most of such researches belong to so-called supervised learning paradigm. Regardless of the modalities of the input signals, ‘labeled data’ with clinicians’ knowledge encoded in are used to ‘train’ such a classifier. The unsupervised counterpart is not very popular in this field yet.

Machine learning-based automatic seizure monitoring is promising yet with intrinsic limitations. A machine learning algorithm learns rules from data automatically and evolves as more data is fed in, so it is a straightforward choice suitable for automatic seizure monitoring. Such algorithms embeded in medical instruments and mobile devices have the potential to reduce the misdetection of seizures and improve the overall efficiency of patients’ medical care. Different from other fields like computer vision, high-quality data are limited in this field because only experts with domain knowledge are capable of labeling bio-signals having high inter- and intra-variability. Inter-observer variability between experts may be considerable. Furthermore, using only information in bio-signals is deemed insufficient for seizure detection and the diagnosis of epilepsy syndrome. For some specific types of seizures, the associated change in bio-signals could not be perceivable. On the contrary, epileptiform discharges are not always recognized as a seizure. The ‘rules’ learned by machine learning algorithms are often only statistically significant relations with poor interpretability, rather than confirmed medical knowledge. Automated decision making will also cause ethical issues and there is a regulation bill requiring the right to an explanation and an option of not being subject to such an automatic decision making procedure has come into force recently (EU GDPR) [22]. 

It should be noted that although it is self-evident that epileptic seizures can be detected. There have been doubts about whether the onset of a seizure can be anticipated [23]. In recent years, many research groups [20,24] have reported their work on seizure predictions. In 2014, the Mayo Clinic and the University of Pennsylvania launched two competitions looking for robust seizure detection and prediction algorithms and released datasets comprised of electrocorticograms (ECoGs) collected from the cortex of canines and humans. Using SVM, random forest and other machine learning techniques, the participants achieved high sensitivity and low false alarm rate on these high-quality datasets [24,25]. A robust automatic seizure/prediction detection algorithm may aid efforts to a closed-loop warning/treatment system. Although there are still no such algorithms that are widely accepted and acknowledged, an implantable closed-loop treatment system aiming for medically intractable refractory partial epilepsy had been approved by the U.S. FDA in 2013. The system monitors and analyses the intracranial electroencephalographic activities of the patient. Neuro stimulus therapy is applied when the approaching of an epileptic seizure is forecasted by the algorithm to terminate the onset and propagation of it. Despite the fact that significant reduction of seizures is observed, complications in patients received implant surgery are also reported [26].

Data analytics of the physiological signal and neuro-images acquired by different diagnostic techniques could provide opportunities for a deeper understanding of the underlying mechanism of epilepsy and enrich the state-of-the-art medical infrastructure. Features extracted from physiological signals which can solve selectivity-invariance dilemma, which means the features can discriminate data segments recorded during ictal period from that recorded during inter-ictal period while the intra- and inter-individual variability does not lead to misclassification, is also crucial for a closed-loop warning/treatment system with high sensitivity and minimum false alarm rate.

## 2. Complexity in Epileptic Seizure Monitoring

When researchers from clinical side talk about ‘complexity’ in epilepsy, much more attention is likely to be paid to highly varied clinical manifestations, etiology, patterns of propagation and the evolution of epilepsy with aging, etc., while researchers from the engineering side use ‘complexity’ to refer to untapped information contained in medical images and electrophysiological recordings. Although clinicians interpret these data in their own way, usually with the aid of experience and medical knowledge, experts on the engineering side do not always place a priority on interpretability of their methods. Instead, inspired by complexity theory, they take the human body as an extremely complex system getting input from environment and adapting itself, while the medical images and physiological signals are just observable and measurable ‘states’ or ‘output’ of this system. Direct treatment implications and anticipation of prognosis are not necessary for them. However, exploring the complexity of these signals in a different perspective may shed new light on the analysis of these data. Different methods are utilized to tackle the complexity in physiological signals, mainly for epileptic seizure detection. Among them, three subjects as fountainheads, e.g., non-stationary signal processing, nonlinear dynamics and network science, can be roughly identified. Non-stationary signal processing is the most straightforward methodological source. And nonlinear dynamics and network science influence this field in a more heuristic and subtle manner. Methods originate from more than one of these three subjects could be adopted in research. Since these three subjects provide independent perspectives, we organize the material along this framework.

Since the physiological processes are confirmed to be nonlinear and non-stationary, non-stationary signal processing techniques are the most intuitive choice for such problems. Compared with traditional time-domain statistical methods and frequency-domain methods which provide averaged information, non-stationary signal processing methods such as short-time Fourier transform, time frequency analysis, wavelet transform [27] and model-based analysis has the advantages of representing and capturing transient anomalies. 

Various measures of the complexity of a function stem from the research about nonlinear dynamics are used to discriminate physiological processes under different pathological conditions [28]. These methods propose a different paradigm and independent information compared to that acquired by classical spectral analysis and non-stationary signal processing techniques. In classical spectral analysis, a signal is treated as a function and is correlated with harmonics of different frequencies. Coefficients are thus employed to measure the ‘intensity’ of different frequency components in this signal. While for most of non-stationary signal processing methods, similar operations were performed on different time scales and different resolution levels. And the signal is not necessarily to be correlated with trigonometric functions. Instead, more functions with desired characteristics can be used (wavelets). Furthermore, in-situ process without the need of another function was developed [29]. For all these methods, only homogenous descriptions (a set of weighted and mutually independent feature vectors, describing homogenous properties of interest) can be expected because the trigonometric functions and ‘wavelets’ are served as the basis of $L^{2}\left(R\right)$ space. The internal correlations and similarities of a signal are ignored. Different from the ‘decomposition-and-superposition’ paradigm where data segments are treated, in some way, individually first, and then accumulated, correlations and similarities in a function (signal) can be reflected by such measures derived from nonlinear dynamics, so these measures of complexity can provide information sometimes unattainable by other methods.

The research on epilepsy is also inspired by network science [30]. Complex networks are graphs with huge numbers of nodes and edges connected. The connection can be direct or indirect, weighted or unweighted, and could even evolve with time. The ‘nodes’ and ‘edges’ are abstractions of ‘entities’ and ‘relations’ in the real world. With a graph, the relations between entities of interest could be modeled. However, network science not only pays attention to the topology of a graph, which is the focus of classical graph theory, but also cares about the dynamics and the equilibrium of a network and the control strategy for different purposes. The influence of network science proceeds along two theoretical approaches. Due to the anatomical basis of human’s brain, a complex network is an analogy of the human brain on many important aspects. The use of mature methods developed in network science to depict the topology of an anatomical brain network may help explain the structural and functional abnormity of the brain of patients with epilepsy. Another approach is relatively suggestive. Graph and time series are two distinct kinds of mathematical objects. Heuristic transformation rules are proposed to set up a bridge between them. When physiological signals are transformed into graphs, matured methods in network science can be applied directly. For such a graph, periodicity is ill-defined. Instead, topological properties describe the physiological signal in a very different way.

### 2.1. Nonlinear Signal Processing

Stationarity of the signal is usually an assumption that oversimplifies reality. The physiological signals are non-stationary signals, whose frequency components will vary under different pathological conditions, including the onset of seizures. In classical Fourier analysis, a section of the time-domain signal is treated as one cycle of a periodic function. With certain smoothness and continuity guaranteed, the function can be decomposed into a series of weighted trigonometric functions. A frequency-domain representation is thus obtained. Usually, real world signals possess ‘good’ continuity, which implies fast decay of the coefficients. Dominant frequency component can be identified. But most of the physiological signals like EEG, EMG are not periodic signals. ECG is pseudo-periodic but still not a strictly periodic signal, so no proper length of time-domain signal can be determined from the signal itself to perform the periodic extension, a step served as conceptual premise before Fourier transform but do not need any actual computations. The results of Fourier analysis may thus alter when the length of data segments, decided artificially, changes. Furthermore, the results could be unstable even with fixed-length data segmentation, due to the shift of the underlying physiological processes. The non-stationarity of physiological signal is the challenge for automatic seizure detection algorithms.

Gotman was the pioneer in the exploration of an EEG-based automatic seizure detection method. To capture the transient behavior during long-term EEG monitoring, in [17], EEG signals are decomposed in time-domain into half waves, based on morphological characteristics. The half waves are then characterized in terms of its duration and amplitude compared to background activity. Typical spikes in EEG recordings, which are usually accompanied by the onset of epileptic seizures, are thus possible to be recognized using a real-time computerized algorithm. Artifacts reduction and inter-channel relations are also discussed in this work, being unsolved problems in EEG-based automatic seizure detection. The false alarm rate is high [31] because the waveforms of many different types of artifacts and non-epileptogenic EEG burst are quite similar to the waveforms during epileptiform discharge. Visual inspections and validations from clinician are necessary [18]. However, a high false alarm rate may place a heavy burden on clinicians. Artifacts are some of the major reasons for spurious detections and they could also overwhelm rhythmic activity when a seizure is approaching. Artifact reduction helps to reduce the false alarm rate and yield a less contaminated signal. Usually artifacts in EEG recordings are caused by eye blinking, muscle contraction and relative displacement between electrodes and patients. The frequency bands where most of the energy of such noises lies in are sometimes overlapped with EEG sub-bands, so frequency-domain filtering will eliminate both noise and useful signal. Independent component analysis (ICA), adaptive filtering [32] and canonical correlation analysis (CCA) [33] are widely employed for preprocessing of (multichannel) EEG signals.

Short time Fourier transform (STFT) is one of the most popular techniques used for non-stationary signals. The original signal is truncated into smaller slices and windowed, and then discrete Fourier transform is performed on it so the transient behavioral could be revealed. STFT is widely used as feature extraction technique applied to raw EEG recording [34]. Usually the statistical metrics of the coefficients are taken as features and feed into classifier [35].

Islam, Rastegarnia, and Yang [36] used a stationary wavelet transform (SWT), which is translationally invariant, to de-noise single-channel EEG signals. Single channel EEG recordings are segmented and decomposed to eight different resolution levels with separate frequency bands. The wavelet coefficients are compared with a threshold to decide whether a modification is needed. Finally the inverse transform is applied to reconstruct the signal which supposed to be artifacts-free. No additional assumptions about the data are needed for this method, neither over-correction would happen across channels. Except for that signals corrupted by noise should only account for a relatively small portion of all data being analyzed, since the thresholding operation is based on the statistical properties of wavelet coefficients. The algorithms are tested on both synthesized data and real data and improved the performance of seizure detection algorithm. Furthermore, six types of noise templates mimic common artifacts are synthesized in this study to test the performance of noise reduction algorithm. It is suggested that the explicit modeling of specific types of artifacts instead of statistical modeling of general noise, which is widely used in adaptive filtering, may help develop a less ambitious but more effective, application-specific noise reduction algorithm. But there is still potential for the improvement of statistical modeling of artifacts. Wang et al. [37] proposed that the limitations of statistical modeling could lie in the inherent difference between the distributions of real-world noise, which is usually asymmetry, and the Gaussian distribution, the most frequently used distribution. α-stable distribution, a family of distributions wherein Gaussian distribution is a particular case, was used to model the impulsive noise in EEG. α-Stable distributions can be asymmetric and have a heavy tail, so they are more powerful to represent the impulsive noise, which is a small probability event. Using a state space model estimated by particle filters, model adoptsα-stable distribution shows superior performance in comparison with the same model but adopts a Gaussian distribution. 

In addition to noise rejection, wavelet transforms are widely used to extract features from physiological signals. A discrete wavelet transform was used to decompose EEG signals into approximate coefficients and detailed coefficients [38]. Reduced complexity was observed during the ictal period and the decomposition helped improve the overall detection accuracy. Furthermore, in this study, surrogate data analysis [39] confirmed a more significant nonlinearity of EEG signals during ictal period. Subasi and Erçelebi [40] used a lifting scheme to speed up the computation of wavelet transforms, and logistic regression and neural networks served as classifier with compared performances. Wavelet-based methods depict the signal under different time scales and help discover discriminate features which could be veiled in the original signal [41]. Janjarasjitt and Loparo [42] used wavelet-based methods to model the 1/*f* process. A specific spectral exponent reflecting the distribution of spectral components from low frequency to high frequency is estimated by multichannel ECoG data from patients with right mesial temporal lobe epilepsy. An increased spectral exponent which implies a higher level of scale-invariant was observed in epileptogenic zone during ictal stage. Model-based methods are also not limited to noise rejection, and can be generic and parametric [43,44] so their parameters can be discriminated features, or physiology-based, mimicking the normal rhythm and epileptic discharges [45]. 

**Figure 2:**
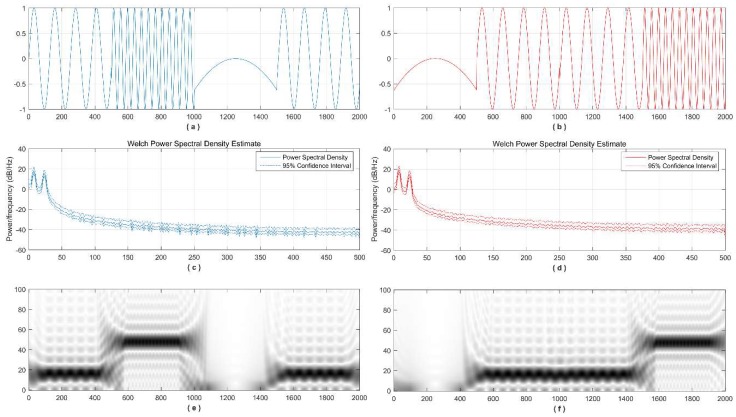
Time-frequency Distribution of Non-stationary Signals: (**a**,**b**) Two signals with frequency components that vary with time; (**c**,**e**) Welch power spectrum density estimation and time frequency distribution of the signal in (**a**); (**d**,**f**) Welch power spectrum density estimation and time frequency distribution of the signal in (**b**).

Joint time-frequency distribution is a powerful tool to adapt to the nature of non-stationary signals. Figure 2 shows that the time-varying frequency components cannot be reflected by spectral analysis but are clear in a time-frequency distribution. One dimensional signals are transformed into a 2-dimensional distribution where for every time point on the x-axis, a distribution of instantaneous frequencies is estimated and plotted on the y-axis. Visual inspection or automatic detection algorithm can be performed on time-frequency distribution (TFD).

Usually, the accuracy (resolution) of frequency components increases with the length of the time window used get longer, at the expense of time localization and vice versa. However, a well-known uncertainty principle, a terminology borrowed from quantum mechanics, asserts that for any function s(t) satisfy the conditions for Fourier transform and normalizing condition that is, $\int_{-\infty }^{\infty }{\left|s\left(t\right)\right|}^{2}dt=1$, then we have that:

$$\left(\int_{-\infty }^{\infty }t^{2}{\left|s\left(t\right)\right|}^{2}dt\right)\left(\int_{-\infty }^{\infty }f^{2}{\left|\hat{s}\left(f\right)\right|}^{2}df\right)\geq \frac{1}{16\pi^{2}}$$

$\hat{s}\left(f\right)$ is the Fourier transform of s(t). This theorem suggests that essential localization of a signal on both time and frequency domain is impossible. Trade-offs should be made depending on the specific requirements for time and frequency resolutions. Time-frequency analysis provides a new idea for feature extraction. Tzallas et al. [46] used different time-frequency analysis to calculate the power spectrum density of EEG signals. Energy fraction measures in specific time-frequency window in TFD are extracted as features and feed into neural networks. High accuracy of detection is achieved. Boashash and Ouelha [47] used modified TFDs and a more comprehensive feature set consists of signal features, statistical features and image features extracted from TFD to handle multichannel EEG data recorded from neonates. A new criterion taking sensitivity into consideration was proposed for feature selection. Reduced computational cost and improved detection performance were obtained together.

Advanced techniques for time series analysis are also used for seizure detection. Celka and Colditz [48] used a computer-aided seizure detection system based on a nonparametric time series modeling method, singular spectrum analysis (SSA). By evaluating the proposed method on both real and synthesized data, a 93% detection rate was obtained and false detection rate was less than 4%. Alam and Bhuiyan [49] transformed the raw EEG into the empirical mode decomposition (EMD) domain. Every raw time series was decomposed into nine intrinsic mode functions (IMFs) and the variance, skewness and kurtosis of each IMF are expected to be different when a seizure happens.

Although EEG reflects the electrical activity on the scalp (sEEG) or in the brain (intracranial EEG) directly, other physiological signals can serve as useful complements. Electrocardiogram (ECG) data is a promising candidate because of it is readily available and its confirmed relation with the autonomous nervous system (ANS). ECG signal contains rich information content, wherein the fiducial points (P wave, QRS complex and T wave) reflects the electrical activity of heart, and the heart rate variability (HRV), defined as the oscillations between consecutive inter-beat intervals, reflects the dynamics of heart activity and is modulated by ANS [50]. The shift in the activity of ANS conducts to heart activity and can be revealed by the analysis on inter-beat intervals. Increased heart rate and occurrence of ECG abnormalities during seizure’s onset have been reported [51]. Simple relative heart rate thresholds can yield promising accuracy [52] while the patients’ age, gender, seizure type and years with epilepsy influence the performance of heart rate based detection [53]. Many research groups have reported their work on seizure detection and prediction utilizing ECG. 

Greene et al. [54] used 41 HRV-based features and linear discrimination model, and achieved considerable detection accuracy in comparison with EEG-based methods. Patient-specific models provide higher accuracy. Qaraqe et al. [55] proposed a method using fused multichannel EEG signals and single-lead ECG signals. A matching pursuit Wigner-Ville distribution (MPWVD) is used to extract features from HRV. Multichannel EEG signals are first enhanced by a common spatial pattern (CSP)-based algorithm and then decomposed by multiresolution wavelet transforms into four sub-bands. The energies of four sub-band signals are computed as features. A data fusion technique reduced the false alarm rate significantly at the expense of a slightly increased detection delay. Fujiwara et al. [20] used generally accepted HRV-based features and multivariate control process to predict the onset of seizure. The definition and other matters need attention can be found in [50] The proposed method was validated on a dataset containing 11 seizures. Ten of the 11 seizures could be predicted prior to their onset, which suggests that some features derived from HRV could serve as precursors of epileptic seizure.

The aforementioned methods investigated the non-stationarity of a signal from a statistical viewpoint. Without the random process serving as a conceptual basis, explicitly or implicitly, one cannot establish the intuition about non-stationarity integrally. Because given a sample of recording, definitely with fixed length and determined content, any analysis performed on this sample will get only one rather multiple results. Recent years, inspired by [56,57], some related research tries to exploit the non-stationarity of physiological signal from a structural viewpoint. Many real world signals are ‘sparse’, which means they can be represented fairly compactly in a certain domain. And the detection of typical pathological condition can be performed on such domains. Nagaraj et al. [58] used atomic decomposition (AD) to represent EEG signals. Orthogonal matching pursuit (OMP) algorithms and four kinds of dictionaries are utilized. The detection of epileptic seizure thus needs no classifiers. Instead, a sole feature measuring the speed of convergence of OMP algorithm is used. Data recorded during seizures converged faster than that recorded during normal condition. In [59], an improved sparse representation–based method was proposed. Multichannel EEG signals were decomposed first and then reconstructed using information contained in physiological significant sub-bands. Together with the following differential operation, artifacts were supposed to be eliminated drastically. The covariance matrices of multichannel EEG signal epochs are computed and Log-Euclidean Gaussian Kernel was adopted to map the covariance matrices into a Reproducible Kernel Hilbert Space (RKHS) so that sparse representation can be applied. The detection of seizure needs no classifier either, a comparison about the residual errors served as the sole feature. The proposed method shows competitive accuracy and low false alarm rate. 

It should be noted that, except for exploring the time-varying characteristics in signal level or feature level, temporal information can be explored at the classifier-level. Although this line of thinking is relatively scarce in the field of automatic epileptic seizure detection, [60] provided an example. A mature technique, dynamic time wrapping (DTW), measuring the similarity between two feature/data sequences with variable length, was used to modify the kernel of support vector machine (SVM). The proposed algorithm is patient-independent. Fused with a modified SVM, a comprehensive improvement was achieved compared with using only static RBF-based SVM. 

Deep learning is a revolutionary paradigm which has overwhelmed the whole machine learning community, especially in the field of computer vision and natural language processing where great success has been achieved. And the influence of deep learning has spread to epileptic seizure monitoring. Thodoroff et al. [61] developed a recurrent neural network framework taking image representation of multichannel EEG data as input for automatic seizure detection. Significant higher sensitivity and lower false alarm rates in comparison with state-of-art algorithms across all patients were obtained. It is worthy of notice that the proposed deep learning framework works more robustly under missing channel conditions than the compound of handcraft features plus SVM. Acharya et al. [62] used a 13-layers deep convolutional neural network (DCNN) to perform computer-aided seizure detection. However, the data used in this research was limited and the amount of parameters in this DCNN is much larger than the number of data, so an extreme over-parametrization was conducted. Seizure prediction leveraged by deep learning has also been reported [63]. Representation learning methods for feature learning are also investigated [64]. Table 1 summarizes these studies.

### 2.2. Nonlinear Dynamics

Nonlinear dynamics is a subject that studies the evolution of a system whose output is not proportional to its input. Differential equations are good mathematical abstractions for dynamic systems. Feedback mechanisms widely exist between the input and output of dynamic systems. With feedback, the evolution of the system could become extremely complex and unpredictable, even though the differential equations governing the system are formally very simple [65,66]. Most of the real dynamic systems in nature are, for all their noteworthy details, nonlinear. Since the physiological processes are relatively stable on large time-scales but unpredictable on short time-scales, which is similar to nonlinear dynamics to some extent, researchers have started to view the human body a complex, nonlinear system in which many of the state variables and their interactions are often unobservable. Physiological signals are observable quantities associated with some state variables, reflecting the system’s response to external stimuli. It is thus possible to show different dynamical characteristics on observable physiological signals of healthy and diseased people. In that way nonlinear dynamics are inspiring research on epilepsy and other diseases.

‘Entropy’ and ‘dimension’ are widely used in this area to measure the ‘complexity’ of physiological signals, in signal level or feature level. However, different entropy measures have different meanings in terms of their theoretical roots [67,68]. Among them, sample entropy [28], approximate entropy [69], multi-scale entropy [70] and distribution entropy [71] were developed. Mandelbrot developed fractal theory [72], a counterintuitive but powerful method to describe the irregularities of an object. Correlation dimension, fractal dimension and largest Lyapunov exponent etc. are widely used to describe the degree of irregularity, and are translated in signal processing to estimate the irregularity of a signal’s waveform or energy distribution. Usually, the interpretations of the results, which always need the context to be justified, are focused on by the researchers aiming to explore the differences hidden in the data across different conditions. However, the consistency of the results should still be a concern. Since entropy measures and dimension estimations are supposed to have small intra-class variation and remarkable inter-class variation an automatic detection algorithm based on such metrics can be anticipated. 

According to [41], EEG signals can be decomposed up to four levels and reconstructed signals approximately correspond to five EEG sub-bands. Correlation dimension and largest Lyapunov exponent (LLE) are calculated on each sub-band signal. These parameters only show differences with statistical significance in sub-band signals rather than the original signals. 

Approximate entropy [69] is one of the most frequently used measures in epilepsy research. It can discern the changing complexity of a dynamic system with relatively few observations (data points). Heuristically, approximate entropy estimates the probability or tendency that patterns close to each other will remain close to each other. In [38,73], approximate entropy is calculated at the signal level and feature level as a discriminative parameter. Liang, Wang, and Chang [74] reported that the combination of approximate entropy and spectral features provides robust seizure detection. It was also found the better ability of approximate entropy to discriminate between ictal and inter-ictal EEG recordings. Guo et al. [73] used multiwavelet transform to decompose EEG signals into sub-bands and also approximate entropy are estimated on sub band signals respectively.

Costa, Goldberger, and Peng [70] introduced multiscale entropy (MSE), a modification of sample entropy capable of accounting for the correlations existing on multiple timescales. The key is to perform the moving average (coarse grain procedure) to the raw time series before the estimation of sample entropy, so the entropy measure is supposed to reflect the complexity of the raw time series in different resolution levels. The coarse grain procedure inspired a lot of derivatives of existing entropy measures. Conigliaro, Manganotti, and Menegaz [75] investigated the potential of multiscale sample entropy in seizure detection whereby the ‘multiscale’ analysis is done with a stationary wavelet transform instead of a simple moving average with multiple window lengths. It was found that the sample entropies in the δ and γ bands account for the main changes of signal structure during seizures, similar to the discovery in [41]. Labate et al. [76] proposed multiscale permutation entropy (MPE) and justified its ability to separate patients with epilepsy from healthy controls. Li et al. [68] proposed a new complexity measure, distribution entropy and compared it with the well-studied irregular measure, sample entropy. It was found that for a database comprised of short-length EEG, distribution entropy are independent from parameter setting while sample entropy could be invalid under improper parameter setting. The results are stable under different protocols, so the consistency and reproducibility of these two measures are validated experimentally. Kannathal et al. [77] compared four kinds of entropies measures with respect to their ability to seizure detection, including spectral entropy, Renyi entropy, Kolmogrov-Sinal entropy and approximate entropy. All the entropy measures showed significant lower values on epileptic group compared to control group.

Polychronaki et al. [78] evaluated the accuracy and three kinds of algorithms estimating the fractal dimension (FD), Katz’s algorithm, Higuchi’s algorithm and k-nearest neighbor (k-NN) algorithm. Only the k-NN algorithm showed consistent changes approaching a seizure’s onset. Furthermore, with a synthetic signal whose fractal estimation can be derived analytically, the accuracies of estimations done by different algorithms and the noise sensitivity were compared systematically, whereby k-NN algorithms also outperformed the former two, most commonly adopted algorithms. Different from most of the aforementioned entropy measures, state-space reconstruction, which can be seen as down-sampling procedure practically while the rigorous theoretical guarantee is established by [79], is not necessary for these algorithms, so a shorter time window can be used and provide better time resolution.

Jouny and Bergey [80] systematically evaluated the ability of spectral measures and complexity measures on seizure detection. A total of 18 different measures were applied to intracranial EEGs recorded from 45 patients with partial seizures. An increased complexity was found to be a possible precursor. However, the class of measures (spectral or complexity) does not guarantee their accuracy, since the conceptual distinction of the class of measures was mainly determined by domain knowledge from other disciplines. A multimodal method may help understand the characteristics of partial seizures.

ECG is a clinically relevant modality for seizure detection. Poincare plot, a visualization tool for HRV, is widely used in related research. Consecutive inter-beat-intervals are plotted one against another on 2-D plane or 3-D space. Larger constant lags are permitted [81]. Poincare plots can also be recognized as a kind of nonlinear analysis based on state-space reconstruction. However, the most frequently used descriptors of Poincare plots are equivalent to simple statistics of the original time series [82] so it seems they cannot truly reflect the ‘nonlinearity’ of the data. A novel descriptor was been proposed [83], but its application in seizure detection is scarce in the literature. Jeppesen et al. [84] proposed modified CSI, a modified descriptor introducing nonlinearity in its definition. The modified descriptor can reflect the abnormal increase of sympathetic activity better. A fixed threshold is used for detection and the modified CSI achieves 100% sensitivity on 13 of 15 patients having focal seizures. Table 2 summarizes these studies.

### 2.3. Network Science

Research into network science was initiated in the late 1990s. Watts and Strogatz described small-world networks with both local connectivity and short average path length [85]. Brabasi and Albert constructed scale-free networks with power-law degree distributions [86]. Subsequent studies have revealed that many natural and social phenomena exhibit small-world and scale-free characteristics. In reality, some of the characteristics of such connected entities can be reflected by topological metrics of the corresponding graphs, including: degree, centrality, average path length, clustering coefficient, etc. 

There are numerous neurons in the brain network. The function of individual neuron cells is relatively simple, while large numbers of hierarchical, interconnected neurons constitute the structural basis of brain functions. The functional and structural organization of the brain network could be altered by patient’s condition. These anomalies can be discovered on different modalities, including EEG, MEG and fMRI [87]. The onset of an epileptic seizure is often accompanied by excessive synchronous discharge of neurons, which can be reflected by EEG. As a model complex networks could be an analogue of real human brains due to their similar behavior [88,89]. For example, the strong local connectivity and short average path length of small-world networks are similar to the human brain in regard to information processing, and the topological characteristics of a graph can explain the synchronous behavior on complex dynamic network, which implies a new direction to uncover the mechanism of epileptic seizures. 

However, there is a gap between real anatomical brain networks and complex networks as models. Some researchers have incorporated anatomical information and observed data to construct complex brain networks, in which the nodes have mappings into certain encephalic regions while the connections between the nodes are determined by observed data. These models can reflect the functional and structural changes of human brain with the help of the physiological signal and neuroimaging while avoid the loss of the interpretability of the model. The other paradigm was launched in a more heuristic manner. Algorithms converting time series into graphs are proposed. Mature graph theory tools can be applied to characterize a time series from a new perspective. 

Ortega, Sola, and Pastor [90] generated a minimal spanning tree (MST) from the correlation matrix whose entries are Pearson correlation coefficients between pairwise multichannel ECoG recordings and identified the so-called local crucial node (LCN) which has largest average correlations with its first neighbors as an indication for epileptogenic zone localization. Ponten, Bartolomei, and Stam [91] used synchronization likelihoods instead of Pearson correlation coefficients to calculate a similar square matrix and then transform it into a binary graph with adaptive thresholds. Larger clustering coefficients and higher characteristic lengths were observed on data collected from patients with temporal lobe seizures. The brain network seems to exhibit small-world properties during ictal period of seizure.

A functional brain network possesses a more random topology during non-seizure periods. Wilke et al. [92] use a directed transfer function (DTF) to generate the causal network and identify the activated nodes from three physiologically-sound sub bands of intracranial EEG recordings and betweenness centrality of activated nodes, defined as the ratio between the number of shortest pass through a specific node to the number of shortest paths of the whole network, was found to be related with ictal activities. This research backs the recently proposed opinion that the ictal activity should be recognized as a network disorder instead of generating from isolated foci. Douw et al. [93] found increased theta band connectivity in MEG is related to a higher number of seizures in patients with brain tumor whereby the functional connectivity is characterized by phase lag index (PLI). Graph theoretical analysis on the networks constructed from PLIs between different channels of ECoG reveals that topological characteristics (clustering coefficient, path length and small world index) of such abstract networks are negatively correlate with the duration of temporal lobe epilepsy (TLE) [94]. Yasuda et al. [95] reported the first evidence about altered topological organization in TLE through the analysis on MRI data. Pearson correlation coefficients between different regions of the brain with interference factors removed represent the structural connectivity. Decreased global efficiency, a metric defined as the inverse of the shortest path length of the structural network, is observed in both right TLE and left TLE groups. Increased local efficiency was only observed from right TLE group.

Zhang and Small [96] were the first to bridge time series to complex networks. They found pseudo periodic time series with different dynamics, when being transformed to a complex network, exhibit distinct topological structures. Lacasa et al. [97] introduced visibility algorithm constructing a graph remains invariant under affine transformations of the original time series. Zhu, Li, and Wen [98] proposed a modified algorithm, called fast weighted horizontal visibility algorithm (FWHVA). Mean length of the transformed graph as feature is efficient to distinguish seizure from healthy. Wang and Meng [99] constructed a functional brain network with MEG data. The individual nodes correspond to specific brain areas while the connections between nodes are determined by the degree of phase synchronization. Significant differences between the clustering coefficients and shortest path length of the functional brain network of patients with epilepsy and healthy control are confirmed. Diykh, Li, and Wen [100] constructed weighted undirected networks from feature vectors instead of raw data. The modularity of transferred network outperformed other network characteristics. Wang et al. [101] studied the EEG seizure patterns’ influence on detection performance. A visibility graph algorithm and two derivatives are applied on EEGs recorded from epileptic patients with intellectual disability. Features based on degree distribution were found efficient in distinguishing seizure EEG from background EEG and improved the detection accuracy on intellectually disabled patients whose seizure patterns are highly varied. Table 3 summarizes these studies.

## 3. Comparison and Discussion

**Figure 3:**
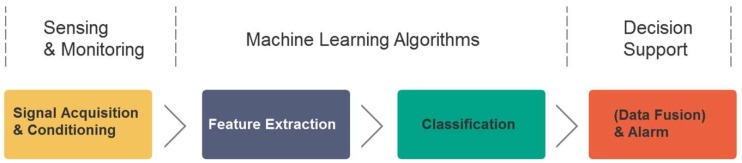
Automatic seizure monitoring by machine learning techniques.

Generally, complexity science has provided new insights to epilepsy research. Progress in the monitoring of epileptic seizures more often comes from the engineering side. The concepts and methods derived from nonlinear signal processing, nonlinear dynamics, and network science are employed as feature extractors. Individual features are not necessarily to be physiologically interpretable, nor highly discerning. Moreover, in the field of machine learning, it is very common to combine multiple low-level features to solve a specific classification task. Several studies about epileptic seizure detection have shown that the fusion of multiple features almost always improves the classification accuracy [102,103]. The schematic diagram of the paradigm a typical machine learning algorithm abides is shown in Figure 3. 

In fact, machine learning is the primary means by which scientists and engineers tackle the problem of detecting/predicting seizures [104,105]. Many of the aforementioned studies, after extracting the features that reflect the complexity of the electrophysiological signal, utilize machine learning models including Artificial Neural Networks (ANN) and Support Vector Machines (SVM). Generally speaking, machine learning is crucial in data-driven decision making, including today’s epileptic seizure monitoring. There is no doubt that the application of confirmed machine learning algorithms in clinical practice is capable of being beneficial to both patients and clinicians. And machine learning will bring about the influence far beyond barely interpreting medical data ‘as good as’ or ‘even better’ than clinical experts.

However, there is a gap between physiological interpretability and universal machine learning paradigm. How these low-level features interact with each other in the machine learning model is not obvious. Despite the superior performance of these models with respect to traditional statistics, the limited interpretability of machine learning models also raises concerns about safety and ethical issues in fields including but not limited to medicine. Furthermore, although there are many ways to evaluate the generalization ability, the ability to adapt to samples obeying the same underlying mechanism but beyond the training set, of a machine learning model, the datasets used in related research are still in deficient in some aspects. Many research groups used non-public data so the results are not reproducible by other groups. The comparison between different methods is impossible without a shared, high quality database. There are several open source databases in the literature: Bonn seizure database [106], MIT-CHB [107], Frieburg epilepsy database, Flint Hills, Epilepsiae [108], MayoClinic [109], Temple University EEG Corpus [110]. Table 4 summaries these public databases. Longitudinal data collected from a population covering a large number of different epilepsy syndromes, different seizure types, and providing clues about the development of epilepsy with various therapies, is still relatively scarce. The lack of database limits the research along this direction. 

In recent years, due to the development of wearable devices, researchers have attempted to use new modalities that do not directly relate to the changes of brain function, such as motion signal (acceleration) [111], electromyography (EMG) signal and electrodermography (EDG) signals to monitor patients with specific seizure types. Of course, ECG is also involved in such wearable sensor systems. The miniaturization of inertial measurement units (IMU) being comprised of accelerometer, gyroscope and/or magnetometer, has paved the way for cost-efficient movement monitoring [112]. E-textiles [112,113], capacitive sensing [114], polymer materials like CNT/PDMS [115] and micro-needle array [116] are used as wearable counterparts of pre-gelled Ag/AgCl electrodes for surface bio-signal acquisition [117,118]. These signals can thus be non-invasively monitored for a long period of time by wearable sensor systems. Although not directly related to the pathologies or in high priority for diagnosis according to the traditional clinical workflows, some indicative clinical manifestation of certain seizure types can be detected from these modalities [19]. To date, most of these portable wearable systems aim for anomalies detection and early warning only. A complete closed-loop consists of not only a sensing module but also an online characteristic events detection algorithm, maybe even associated medical interventions after alarms being raised.

A wearable sensor system with robust algorithm detecting seizures of certain types with high sensitivity and tolerable false alarm rate can help fill up the vacancy of daily used seizure monitoring device and reduce the miss detection rate in clinical scenarios. Therefore, it is prosperous to establish new approaches based on these new modalities for the monitoring of seizures in unstructured environments. Several research groups have reported related work where hybrid features of the signal, including spectral features and complexity features, are commonly used.

Usually, electromyography signals and movement are recognized as two major sources of artifacts in EEG signal analysis. However, for some typical seizure types, like generalized tonic-clonic seizure (GTCS), specific muscular activities and body movements are important clinical manifestations. Since EMG and motion signals can be collected by wearable sensor systems in an unobtrusive manner and long-term monitoring is more applicable, the investigation on the value of acceleration signal and EMG signal in seizure detection has become an emerging field of study over the last decade. Wavelet-based time-frequency analysis [119] of data from 3-D acceleration sensors placed on the limb and sternum of patients having myoclonic seizures yielded 80% sensitivity while false alarms are likely to be triggered by normal movements compared to tonic or clonic movements. Poh et al. [120] used wearable sensors to monitor the electrodermal activity and the movement of patients. Hybrid features (19 features from time-domain, frequency domain and features derived from nonlinear dynamics) are extracted and feed into SVM. EDG signals improved the sensitivity under the same specificity level. In [121] a seizure detection algorithm based on the analysis on sEMG signal showed high sensitivity and specificity to GTCS, and it was reported that false alarms were not triggered by other types of seizures. Milosevic et al. [122] investigated the detection of tonic-clonic seizure in pediatric patients with sEMG sensors and acceleration sensors attached to the patients’ arms, in which the sEMG sensors aim to detect the tonic phase of a seizure and acceleration sensors are aimed to detect the clonic behavior of patient. Features from different domains were extracted from acceleration signal and sEMG signal and feature selection was performed based on mutual information criteria. Goldenholz et al. [123] found reduced blood oxygen saturation (SpO_2_) could be associated with the termination of seizures. In this study, a simple threshold rather than sophisticated machine learning technique is capable of detecting up to 94% of generalized seizures in all evaluable data. SpO_2_ is also a modality suitable for long-term low-burden monitoring. Table 5 provides a non-exhaustive collection about related research.

Analyzing physiological signals from a complex point of view can also help promote our understanding about epilepsy. Complexity-based methods can also identify interpretable precursors of the clinical onset of epileptic seizures [84]. Sometimes a single precursor is indicative enough and no machine learning techniques are needed [38]. The shift of the underlying dynamics of physiological process during ictal period was revealed. Reduced complexity and increased order of the functional brain network caused by seizures were discovered [41,100]. Since the real communications between trillions of neurons are undetectable, functional brain networks inspired by network science possess unique value in neuroscience. In this direction, new schemes to localize epileptogenic zone were also developed.

## 4. Conclusions

Complexity science explains a wide range of natural and social phenomena. A shared language is usually impossible when different disciplines use their methods to study complexity. Different approaches also have different theoretical roots. Fractal theory comes from the study of geometry. The flourishing status of nonlinear dynamics would not be without the achievements of information theory and differential equation theory. Network science stems from graph theory and cybernetics. Despite the lack of clarity about the boundaries of complexity science, the methods provided are powerful and the perspectives offered are unique and irreplaceable by other methods, in epilepsy research.

Research about epileptic seizure detection/prediction is restricted by the limited amount of available public, high quality data. Many research groups conduct their studies on private datasets. Large-scale and high-quality public available datasets will facilitate the progress in this direction, making the comparison and reproduction of different methods easier and finally, promote the research results into clinical practice.

In addition, the advances in wearable technology in recent years have inspired researchers to use new modalities for the detection of seizures of specific types, although in principle it is difficult to detect epileptic seizures without relevant clinical manifestations motion signals, EMG signals and EDG signals, this direction is still a prosperous research avenue. Currently, most patients with epilepsy live in developing countries, where they suffer from a lack of medical resources. For patients living in developed countries, long-term treatment and monitoring in hospitals are still impractical and could severely reduce their quality of life (QoL). Non-intrusive monitoring with wearable devices is expected to play a role in non-hospital settings in the absence of caregivers. Sudden unexpected death in epilepsy (SUEDP) is the leading cause of death in patients with epilepsy and many patients die from drowning caused by the onset of seizures while taking a bath. The abnormal event can be detected by a wearable device embedded with robust algorithms (which may be also patient specific), so a healthcare worker or family member could response in a timely way. Furthermore, epileptic seizures are multi-modal in nature, so the accumulation of data of different modalities may also contribute to the understanding of seizures. Major obstacles lie in the dilemma between high sensitivity of anomalies and low false detection rate since an intolerable false alarm rate bears a heavy burden on medical infrastructure and limits its popularization.

## Figures and Tables

**Table 1:** Epileptic Seizure Monitoring using Non-stationary Signal Processing (Sen: Sensitivity. Spec: Specificity. FDR: False detection rate. “*” indicates nonpublic dataset. LDA: linear discriminative analysis. LR: logistic regression. Bonn, Freiburg, EPILEPSIAE:abbreviations of datasets).

Author (Year) [Reference]	Signal	Dataset	Features	Classifier/Methods	Results
M. Roessgen et al. (1998) [46]	EEG	EEG from 2 babies *	Model parameters	--	Highly reduced FDR
Patrick Celka et al. (2002) [48]	EEG	EEG from 4 babies	Singular values and minimum description length	Simple thresholding	>93% detection rate and <4% FDR
Greene et al. (2007) [55]	ECG	ECG signal from 7 neonates having 520 seizure events *	Time domain, frequency domain and time-frequency domain features	LDA	62.2% Sen, 71.8% Spec
A. T. Tzallas et al. (2009) [47]	EEG	Bonn	Power spectral features from 12 time-frequency distributions	ANN	89–100% Sen, 89.1–100% Spec across different tasks
S. M. Shafiul Alam et al. (2013) [49]	EEG	Bonn	Higher order statistics in EMD domain	ANN	100% Acc
Nagaraj et al. (2014) [58]	EEG	826 h EEG from 18 full-term neonates with 1389 seizures *	Relative structural complexity	Simple thresholding	0.91 Area under the curve (AUC)
K. Samiee et al. (2015) [35]	EEG	Bonn	Spectral coefficients with their statistical values	Naive Bayes/LR/SVM/K-NN/ANN	98.8% Sen, 97.2% Spec, 98.3% Acc
Shasha Yuan et al. (2016) [59]	EEG	Freiburg	Residual error after reconstruction	Simple thresholding	95.11% Sen, 98.78% Spec
Boashash et al. (2016) [47]	EEG	EEG from 36 sick newborns *	Feature set extracted from TFD	Random forests/SVM/ANN	86.61% accuracy
Marwa Qaraqe et al. (2016) [55]	EEG/ECG	EPILEPSIAE	Skewness of original signal, mean and standard deviation of TFD	SVM	100% Sen with varied false alarm rate due to different specifications of model
Fujiwara et al. (2016) [20]	ECG	ECG signal from 14 patients (metadata reported) *	HRV-based features	Multivariate process control	91% Sen, 0.7 times/h FDR (Prediction)
P. Thodoroff et.al (2016) [61]	EEG	CHB-MIT	Image representation of EEG integrating spatial information	Recurrent neural network	Higher Sen, lower FDR
R. Ahmed et al. (2017) [60]	EEG	261 h EEG from 17 neonates with 821 seizures *	Time and frequency domain features and entropy measures	SVM-(modified kernel)/RBF-SVM	82.6% Sen and 90% Spec after post-processing
U. R. Acharya et al. (2017) [62]	EEG	Bonn	--	DCNN (13 layers)	95% Sen, 90% Spec, 88.67% Acc
Ye Yuan et al. (2017) [64]	EEG	CHB-MIT	Short-time Fourier transform	mSSDA/softmax	93.82% Acc
I. K. Kornek (2018) [63]	EEG	Intracranial EEG from 15 patients with 2817 seizures	Time-frequency representation	Deep neural network	42% Sen surpassing random predictor

**Table 2:** Epileptic Seizure Monitoring using Nonlinear Dynamics (Sen: Sensitivity. Spec: Specificity. FDR: False detection rate. “*” indicates nonpublic dataset. CD: correlation dimension. LLE: largest Lyapunov exponent. ANFIS: adaptive neuro fuzzy inference system).

Author (Year) [Reference]	Signal	Dataset	Features	Classifier/Methods	Results
N. Kannathal et al. (2005) [77]	EEG	Bonn	Kolmogorov entropy/Spectral entropy/Renyi entropy/Approximate entropy;	ANFIS	91.49–93.02% Sen
H. Adeli et al. (2007) [41]	EEG	Bonn	CD/LLE	--	CD is distinct in beta and gamma band while LLE is distinct in alpha band
Ocak et al. (2009) [38]	EEG	Bonn	Approximate entropy	Statistical analysis	96% accuracy
Lin Guo et al. (2010) [73]	EEG	Bonn	Approximate entropy	ANN	98.27% accuracy
Polychronaki et al. (2010) [78]	EEG	553 h EEG from 8 patients with 55 seizures (metadata reported) *	Fractal dimension	Simple thresholding	100% Sen, 0.42 times/h FDR
Sheng Fu Liang et al. (2010) [74]	EEG	Bonn	Approximate entropy and frequency domain features	LDA/SVM/ANN/	97.82–98.51% accuracy, (seizure/non-seizure)
C.C. Jouny et al. (2012) [80]	EEG	Intracranial EEG from 45 patients	Bundles of frequency-based and complexity-based features	--	Gabor atom density, Lempel-Ziv complexity, Higuchi fractal dimension, high frequency activity, sample entropy were more reliable to assess early seizure onset
Labate et al. (2013) [76]	EEG	EEG collected from 22 patients and 35 healthy controls *	Multiscale permutation entropy	SVM	77–88% Sen, 55–87% Spec
Conigliaro et al. (2014) [75]	EEG	8 h EEG *	Multiscale sample entropy and spectral features	SVM	89–99% accuracy across 5 patients with TLE
Jesper Jeppesen et al. (2015) [84]	ECG	ECG from 17 patients with 17 seizures	Heart rate variability based features	Simple thresholding	Modified Cardiac Sympathetic Index (mCSI) performs well, 13 of 17 seizures are detected
Yueming Wang et al. (2016) [37]	EEG	CHB-MIT and 331 h EEG from 9 patients with 9 seizures *	Sample entropy and other morphological features	State space model	89% Sen and 0.48 times/h FDR on CHB-MIT database; 100% Sen and 0.08 times/h FDR on private dataset.
P. Li et al. (2016) [71]	EEG	Bonn	Distribution entropy and sample entropy	Statistical analysis	0.93–0.97 AUC for sample entropy; 0.66–0.87 AUC for distribution entropy but with higher robustness for short length data

**Table 3:** Epileptic Seizure Monitoring inspired by Network Science (Sen: Sensitivity. Spec: Specificity. FDR: False detection rate. “*” indicates nonpublic dataset).

Author (Year) [Reference]	Signal	Dataset	Methodology	Results and Discoveries
S. C. Ponten et al. (2007) [91]	EEG	Intracerebral EEG from 7 patients	Synchronization likelihood based abstract network construction	The abstract brain network tends to a more ordered configuration during seizure activities, with higher clustering coefficient and larger shortest path length
G. J. Ortega et al. (2008) [90]	ECoG	ECoG from 5 patients	Minimum spanning tree on correlation matrix deployed as a metric of connectivity	Regions identified by complex network analysis that with higher local synchronization power is related to the development of epileptic seizure
van Dellen et al. (2009) [94]	ECoG	ECoG from 27 patients	Phase lag index is used to construct the functional brain network	Averaged PLI, clustering coefficient are negatively correlated with the duration of TLE.
Linda Douw et al. (2010) [93]	MEG	17 patients and 12 of them at two time points	Phase lag index is used to construct the functional brain network	Altered functional connectivity and less optimal brain network topology in patients. Increased theta band connectivity is related to larger number of seizures
Christopher Wilke et al. (2011) [92]	ECoG	ECoG from 25 patients	Directed transfer function are used to construct the connection of brain network	The betweenness centrality is probably indicative of epileptogenic zone. Such correlations are frequency dependent.
Zhu Guohun et al. (2014) [98]	EEG	Bonn	Mean degree and mean strength	93–100% accuracy across different tasks
C. L. Yasuda et al. (2015) [95]	MRI	86 patients with left TLE, 70 patients with right TLE and 116 healthy controls	Pearson correlations are used to construct the structural brain network	Decreased global efficiency and increased local efficiency were observed in TLE group
W. Beilei and L. Meng (2016) [99]	MEG	MEG from 20 patients and 20 health controls	Phase lag index is used to construct the functional brain network	Frequency-dependent alteration of the metrics of brain network in patients with epilepsy was observed
Diykh et al. (2016) [100]	EEG	Bonn	Modularity, closeness centrality, clustering coefficient, average shortest path length	97% Sen, 99% Spec, 98% accuracy
Wang Lei et al. (2017) [101]	EEG	615 h EEG from 29 patients with 91 seizures *	Degree entropy and six features based on wavelet analysis	38% Sen for combined, higher than 24% when only use wavelet-based features.

**Table 4:** Public-available datasets for the research about automatic seizure monitoring (SF: sampling frequency. ADC: analog-to-digit converter. ECoG: Electrocorticogram).

Database	Basic Description	Metadata	Label	Scale
Bonn Seizure Database	5 groups of single channel EEG recordings. SF is 173.6 Hz and the bandwidth of raw data is 0.53–85 Hz.	5 patients and 5 healthy controls. No more details.	Normal(A,B)/Ictal (E)/Inter-ictal(C,D)	Each group with 100 records and each record with a length of 23.6 s (4096 data points).
CHB-MIT Scalp EEG Database	Multichannel (23, 24 or 26) EEG from 22 patients with SF of 256 Hz and 16 bit ADC. Protected health information (PHI) of patients has been masked.	17 females, ages 1.5–19. 5 males, ages 3–22.	Start time and end time (accurate to second).	24 cases with 664 files in total, among which 129 files contain 198 seizures.
Flint Hills Scientific L.L.C.	Multichannel (48–64) ECoG with SF of 249 Hz.	10 patients	Start time and end time	1419 h, 59 seizures
Freiburg	Multichannel long-term ECoG collected from 21 patients with medical intractable epilepsy by grid-, strip- and depth electrodes. SF is 256 Hz and 16 bit ADC is applied.	Ages (8 males, 13 females), seizure types, durations are available.	Start time and end time (accurate to second).	87 seizures in total. For records contain seizure, at least 50 min pre-ictal data are provided.
European Epilepsy Database	Both surface and intracranial multichannel EEG from more than 250 patients (60 available now) in three centers (Coimbra, Paris and Freiburg). SF ranges from 250 to 2500 Hz.	Clinical patient information and (most of) MRI data.	Well annotated by EEG experts with supplementary metadata.	40,000+ h, 2400+ seizures. For each patient, more than 150 h continuous EEG data provided.
U Penn & Mayo Clinic’s Seizure Detection	Intracranial EEG from 4 dogs and 8 patients suffering from drug-resistant epilepsy. 16 channels and a SF of 400 Hz for dogs. 16–72 channels and SF of 500/5000 Hz for patients.	Gender, age and epileptic zone for patients.	Inter-ictal/Ictal	58,837 clips (25,922 in training set/32,915 in test set). 1 s of length for each clip.
U Penn & Mayo Clinic’s Seizure Prediction	Intracranial EEG from 5 dogs and 2 patients. 16 channels and a SF of 400 Hz for dogs. 15 channels and SF of 5000 Hz for patients.	Gender, age and the arrangement of electrodes.	Inter-ictal/Preictal Events annotated	8002 clips (4067 in training set/3935 in test set). 10 min of length for each clip.
THU EEG Seizure Corpus	Multichannel (24–36) EEG with a SF of 250 Hz.	Patient’s clinical history and medications	Medical records available	16,986 sessions from 10,874 unique subjects

**Table 5:** Application of non-EEG based wearable sensor system in Epileptic Seizure Monitoring.

Author (Year) [Reference]	Modality	Methods and Features	Results and Discoveries
T. M. E. Nijsen et al. (2005) [19]	Acceleration	Modulus of three axis acceleration	Acceleration detected 428/897 seizures along and 10/18 patients’ seizures can all be detected by acceleration
T. M. E. Nijsen et al. (2012) [119]	Acceleration	Model-based match wavelet transform of acceleration	80% Sen, 85% Spec
Ming-Zher Poh et al. (2012) [120]	EDG and acceleration	Hybrid features and SVM as classifier	15 of 16 generalized tonic clonic seizures (GTCS) are detected from >4213 h recordings from 80 patients with false alarm rate about 0.74 times per day
C. A. Szab (2015) [121]	sEMG	Brain Sentinel’s algorithm	20 of 21 GTCS are detected in 11 patients from 1399 h’s recording from 33 patients
Milosevic et al. (2016) [122]	sEMG and acceleration	Hybrid features and SVM as classifier	Multimodal method outperforms any of unimodal methods, 90.91% Sen, 0.45 FDR/12 h

## References

[1] Moshé S.L., Perucca E., Ryvlin P., Tomson T. (2015). Epilepsy: New advances. Lancet.

[2] Kwan P., Schachter S.C., Brodie M.J. (2011). Drug-Resistant Epilepsy. N. Engl. J. Med..

[3] Magiorkinis E., Sidiropoulou K., Diamantis A. (2010). Hallmarks in the history of epilepsy: Epilepsy in antiquity. Epilepsy Behav..

[4] Gastaut H. (1964). A Proposed International Classification of Epileptic Seizures. Epilepsia.

[5] Gastaut H. (1970). Clinical and Electroencephalographical Classification of Epileptic Seizures. Epilepsia.

[6] Merlis J.K. (1970). Proposal for an International Classification of the Epilepsies. Epilepsia.

[7] Commission on Classification and Terminology of ILAE (1981). Proposal for Revised Clinical and Electroencephalographic Classification of Epileptic Seizures: From the Commission on Classification and Terminology of the International League against Epilepsy. Epilepsia.

[8] Commission on Classification and Terminology of ILAE (1985). Proposal for Classification of Epilepsies and Epileptic Syndromes. Epilepsia.

[9] Commission on Classification and Terminology of ILAE (1989). Proposal for Revised Classification of Epilepsies and Epileptic Syndromes: Commission on Classification and Terminology of the International League against Epilepsy. Epilepsia.

[10] Fisher R.S., Boas W.V.E., Blume W., Elger C., Genton P., Lee P., Engel J. (2005). Epileptic Seizures and Epilepsy: Definitions Proposed by the International League against Epilepsy (ILAE) and the International Bureau for Epilepsy (IBE). Epilepsia.

[11] Berg A.T., Berkovic S.F., Brodie M.J., Buchhalter J., Cross J.H., van Emde Boas W., Engel J., French J., Glauser T.A., Mathern G.W. (2010). Revised terminology and concepts for organization of seizures and epilepsies: Report of the ILAE Commission on Classification and Terminology, 2005–2009. Epilepsia.

[12] Fisher R.S., Cross J.H., French J.A., Higurashi N., Hirsch E., Jansen F.E., Lagae L., Moshé S.L., Peltola J., Roulet Perez E. (2017). Operational classification of seizure types by the International League Against Epilepsy: Position Paper of the ILAE Commission for Classification and Terminology. Epilepsia.

[13] Scheffer I.E., Berkovic S., Capovilla G., Connolly M.B., French J., Guilhoto L., Hirsch E., Jain S., Mathern G.W., Moshé S.L. (2017). ILAE classification of the epilepsies: Position paper of the ILAE Commission for Classification and Terminology. Epilepsia.

[14] Spencer S.S. (1994). The Relative Contributions of MRI, SPECT, and PET Imaging in Epilepsy. Epilepsia.

[15] Gottschalk S., Fehm T.F., Deán-Ben X.L., Tsytsarev V., Razansky D. (2016). Correlation between volumetric oxygenation responses and electrophysiology identifies deep thalamocortical activity during epileptic seizures. Neurophotonics.

[16] Macé E., Montaldo G., Cohen I., Baulac M., Fink M., Tanter M. (2011). Functional ultrasound imaging of the brain. Nat. Methods.

[17] Gotman J., Gloor P. (1976). Automatic recognition and quantification of interictal epileptic activity in the human scalp EEG. Electroencephalogr. Clin. Neurophysiol..

[18] Gotman J., Ives J., Gloor P. (1979). Automatic recognition of inter-ictal epileptic activity in prolonged EEG recordings. Electroencephalogr. Clin. Neurophysiol..

[19] Nijsen T.M.E., Arends J.B.A.M., Griep P.A.M., Cluitmans P.J.M. (2005). The potential value of three-dimensional accelerometry for detection of motor seizures in severe epilepsy. Epilepsy Behav..

[20] Fujiwara K., Miyajima M., Yamakawa T., Abe E., Suzuki Y., Sawada Y., Kano M., Maehara T., Ohta K., Sasai-Sakuma T. (2016). Epileptic Seizure Prediction Based on Multivariate Statistical Process Control of Heart Rate Variability Features. IEEE Trans. Biomed. Eng..

[21] Bogaarts J.G., Gommer E.D., Hilkman D.M.W., van Kranen-Mastenbroek V.H.J.M., Reulen J.P.H. (2016). Optimal training dataset composition for SVM-based, age-independent, automated epileptic seizure detection. Med. Biol. Eng. Comput..

[22] EU General Data Protection Regulation. https://www.eugdpr.org/eugdpr.org.html.

[23] Jouny C.C., Franaszczuk P.J., Bergey G.K. (2005). Signal complexity and synchrony of epileptic seizures: Is there an identifiable preictal period?. Clin. Neurophysiol..

[24] Brinkmann B.H., Patterson E.E., Vite C., Vasoli V.M., Crepeau D., Stead M., Howbert J.J., Cherkassky V., Wagenaar J.B., Litt B. (2015). Forecasting Seizures Using Intracranial EEG Measures and SVM in Naturally Occurring Canine Epilepsy. PLoS ONE.

[25] Brinkmann B.H., Wagenaar J., Abbot D., Adkins P., Bosshard S.C., Chen M., Tieng Q.M., He J., Muñoz-Almaraz F.J., Botella-Rocamora P. (2016). Crowdsourcing reproducible seizure forecasting in human and canine epilepsy. Brain.

[26] Morrell M.J. (2011). On behalf of the RNS System in Epilepsy Study Group. Responsive cortical stimulation for the treatment of medically intractable partial epilepsy. Neurology.

[27] Mallat S.G. (2009). A Wavelet Tour of Signal Processing: The Sparse Way.

[28] Joshua J.R.M., Richman S. (2000). Physiological time-series analysis using approximate entropy and sample entropy. Am. J. Physiol.-Heart Circ. Physiol..

[29] Sweldens W. Lifting scheme: A new philosophy in biorthogonal wavelet constructions. Proceedings of the SPIE’s 1995 International Symposium on Optical Science, Engineering, and Instrumentation.

[30] Albert R., Barabási A.-L. (2002). Statistical mechanics of complex networks. Rev. Mod. Phys..

[31] Gotman J. (1982). Autom atic recognition of epileptic seizures in the EEG. Electroencephalogr. Clin. Neurophysiol..

[32] Guerrero-Mosquera C., Navia-Vázquez A. (2012). Automatic removal of ocular artefacts using adaptive filtering and independent component analysis for electroencephalogram data. IET Signal Process..

[33] Urigüen J.A., Garcia-Zapirain B. (2015). EEG artifact removal—State-of-the-art and guidelines. J. Neural Eng..

[34] Yadav R., Agarwal R., Swamy M.N.S. STFT-Based Segmentation in Model-Based Seizure Detection. Proceedings of the Canadian Conference on Electrical and Computer Engineering, CCECE 2007.

[35] Samiee K., Kovacs P., Gabbouj M. (2015). Epileptic Seizure Classification of EEG Time-Series Using Rational Discrete Short-Time Fourier Transform. IEEE Trans. Biomed. Eng..

[36] Islam M.K., Rastegarnia A., Yang Z. (2016). A Wavelet-Based Artifact Reduction from Scalp EEG for Epileptic Seizure Detection. IEEE J. Biomed. Health Inform..

[37] Wang Y., Qi Y., Wang Y., Lei Z., Zheng X., Pan G. (2016). Delving into *α*-stable distribution in noise suppression for seizure detection from scalp EEG. J. Neural Eng..

[38] Ocak H. (2009). Automatic detection of epileptic seizures in EEG using discrete wavelet transform and approximate entropy. Expert Syst. Appl..

[39] Schreiber T., Schmitz A. (1996). Improved Surrogate Data for Nonlinearity Tests. Phys. Rev. Lett..

[40] Subasi A., Erçelebi E. (2005). Classification of EEG signals using neural network and logistic regression. Comput. Methods Programs Biomed..

[41] Adeli H., Ghosh-Dastidar S., Dadmehr N. (2007). A Wavelet-Chaos Methodology for Analysis of EEGs and EEG Subbands to Detect Seizure and Epilepsy. IEEE Trans. Biomed. Eng..

[42] Janjarasjitt S., Loparo K.A. (2015). Examination of Scale-Invariant Characteristics of Multi-channel ECoG Data for Epileptic Seizure Localization. J. Med. Biol. Eng..

[43] Kim H., Rosen J. Epileptic seizure detection—An AR model based algorithm for implantable device. Proceedings of the 2010 Annual International Conference of the IEEE Engineering in Medicine and Biology Society (EMBC).

[44] Wang G., Sun Z., Tao R., Li K., Bao G., Yan X. (2016). Epileptic Seizure Detection Based on Partial Directed Coherence Analysis. IEEE J. Biomed. Health Inform..

[45] Roessgen M., Zoubir A.M., Boashash B. (1998). Seizure detection of newborn EEG using a model-based approach. IEEE Trans. Biomed. Eng..

[46] Tzallas A.T., Tsipouras M.G., Fotiadis D.I. (2009). Epileptic Seizure Detection in EEGs Using Time-Frequency Analysis. IEEE Trans. Inf. Technol. Biomed..

[47] Boashash B., Ouelha S. (2016). Automatic signal abnormality detection using time-frequency features and machine learning: A newborn EEG seizure case study. Knowl.-Based Syst..

[48] Celka P., Colditz P. (2002). A computer-aided detection of EEG seizures in infants: A singular-spectrum approach and performance comparison. IEEE Trans. Biomed. Eng..

[49] Alam S.M.S., Bhuiyan M.I.H. (2013). Detection of Seizure and Epilepsy Using Higher Order Statistics in the EMD Domain. IEEE J. Biomed. Health Inform..

[50] Camm A.J., Malik M., Bigger J.T., Breithardt G., Cerutti S., Cohen R.J., Coumel P., Fallen E.L., Kennedy H.L., Kleiger R.E. (1996). Heart rate variability: Standards of measurement, physiological interpretation, and clinical use. Eur. Heart J..

[51] Zijlmans M., Flanagan D., Gotman J. (2002). Heart Rate Changes and ECG Abnormalities during Epileptic Seizures: Prevalence and Definition of an Objective Clinical Sign. Epilepsia.

[52] Osorio I. (2014). Automated seizure detection using EKG. Int. J. Neural Syst..

[53] Osorio I., Manly B.F.J. (2015). Probability of detection of clinical seizures using heart rate changes. Seizure.

[54] Greene B.R., de Chazal P., Boylan G.B., Connolly S., Reilly R.B. (2007). Electrocardiogram Based Neonatal Seizure Detection. IEEE Trans. Biomed. Eng..

[55] Qaraqe M., Ismail M., Serpedin E., Zulfi H. (2016). Epileptic seizure onset detection based on EEG and ECG data fusion. Epilepsy Behav..

[56] Donoho D.L. (2006). Compressed sensing. IEEE Trans. Inf. Theory.

[57] Candès E.J., Romberg J.K., Tao T. (2006). Stable signal recovery from incomplete and inaccurate measurements. Commun. Pure Appl. Math..

[58] Nagaraj S.B., Stevenson N.J., Marnane W.P., Boylan G.B., Lightbody G. (2014). Neonatal Seizure Detection Using Atomic Decomposition With a Novel Dictionary. IEEE Trans. Biomed. Eng..

[59] Yuan S., Zhou W., Wu Q., Zhang Y. (2016). Epileptic Seizure Detection with Log-Euclidean Gaussian Kernel-Based Sparse Representation. Int. J. Neural Syst..

[60] Ahmed R., Temko A., Marnane W.P., Boylan G., Lightbody G. (2017). Exploring temporal information in neonatal seizures using a dynamic time warping based SVM kernel. Comput. Biol. Med..

[61] Thodoroff P., Pineau J., Lim A. Learning Robust Features using Deep Learning for Automatic Seizure Detection. Proceedings of the Machine Learning for Healthcare Conference.

[62] Acharya U.R., Oh S.L., Hagiwara Y., Tan J.H., Adeli H. (2017). Deep convolutional neural network for the automated detection and diagnosis of seizure using EEG signals. Comput. Biol. Med..

[63] Kiral-Kornek I., Roy S., Nurse E., Mashford B., Karoly P., Carroll T., Payne D., Saha S., Baldassano S., O’Brien T. (2018). Epileptic Seizure Prediction Using Big Data and Deep Learning: Toward a Mobile System. EBioMedicine.

[64] Yuan Y., Xun G., Jia K., Zhang A. A Multi-view Deep Learning Method for Epileptic Seizure Detection using Short-time Fourier Transform. Proceedings of the 8th ACM International Conference on Bioinformatics, Computational Biology, and Health Informatics.

[65] Li T.-Y., Yorke J.A. (1975). Period Three Implies Chaos. Am. Math. Mon..

[66] May R.M. (1976). Simple mathematical models with very complicated dynamics. Nature.

[67] Shannon C.E. (2001). A mathematical theory of communication. ACM SIGMOBILE Mob. Comput. Commun. Rev..

[68] Kolmogorov A.N. (1958). A new metric invariant of transient dynamical systems and automorphisms in Lebesgue spaces. Dokl. Akad. Nauk SSSR.

[69] Pincus S.M. (1991). Approximate entropy as a measure of system complexity. Proc. Natl. Acad. Sci. USA.

[70] Costa M., Goldberger A.L., Peng C.-K. (2002). Multiscale Entropy Analysis of Complex Physiologic Time Series. Phys. Rev. Lett..

[71] Li P., Karmakar C., Yan C., Palaniswami M., Liu C. (2016). Classification of 5-S Epileptic EEG Recordings Using Distribution Entropy and Sample Entropy. Front. Physiol..

[72] Mandelbrot B.B., Wheeler J.A. (1983). The Fractal Geometry of Nature. Am. J. Phys..

[73] Guo L., Rivero D., Pazos A. (2010). Epileptic seizure detection using multiwavelet transform based approximate entropy and artificial neural networks. J. Neurosci. Methods.

[74] Liang S.-F., Wang H.-C., Chang W.-L. (2010). Combination of EEG Complexity and Spectral Analysis for Epilepsy Diagnosis and Seizure Detection. EURASIP J. Adv. Signal Process..

[75] Conigliaro D., Manganotti P., Menegaz G. Multiscale sample entropy for time resolved epileptic seizure detection and fingerprinting. Proceedings of the 2014 IEEE International Conference on Acoustics, Speech and Signal Processing (ICASSP).

[76] Labate D., Palamara I., Mammone N., Morabito G., Foresta F.L., Morabito F.C. SVM classification of epileptic EEG recordings through multiscale permutation entropy. Proceedings of the 2013 International Joint Conference on Neural Networks (IJCNN).

[77] Kannathal N., Choo M.L., Acharya U.R., Sadasivan P.K. (2005). Entropies for detection of epilepsy in EEG. Comput. Methods Programs Biomed..

[78] Polychronaki G.E., Ktonas P.Y., Gatzonis S., Siatouni A., Asvestas P.A., Tsekou H., Sakas D., Nikita K.S. (2010). Comparison of fractal dimension estimation algorithms for epileptic seizure onset detection. J. Neural Eng..

[79] Takens F., Rand D., Young L.-S. (1981). Detecting strange attractors in turbulence. Dynamical Systems and Turbulence, Warwick 1980.

[80] Jouny C.C., Bergey G.K. (2012). Characterization of early partial seizure onset: Frequency, complexity and entropy. Clin. Neurophysiol..

[81] Karmakar C., Jelinek H.F., Khandoker A., Tulppo M., Mäkikallio T., Kiviniemi A., Huikuri H., Palaniswami M. Multi-lag HRV analysis discriminates disease progression of post-infarct people with no diabetes versus diabetes. Proceedings of the 2015 37th Annual International Conference of the IEEE Engineering in Medicine and Biology Society (EMBC).

[82] Brennan M., Palaniswami M., Kamen P. (2001). Do existing measures of Poincare plot geometry reflect nonlinear features of heart rate variability?. IEEE Trans. Biomed. Eng..

[83] Karmakar C.K., Khandoker A.H., Gubbi J., Palaniswami M. (2009). Complex Correlation Measure: A novel descriptor for Poincaré plot. Biomed. Eng. Online.

[84] Jeppesen J., Beniczky S., Johansen P., Sidenius P., Fuglsang-Frederiksen A. (2015). Detection of epileptic seizures with a modified heart rate variability algorithm based on Lorenz plot. Seizure.

[85] Watts D.J., Strogatz S.H. (1998). Collective dynamics of ‘small-world’ networks. Nature.

[86] Albertlaszlo Barabasi R.A. (1999). Emergence of Scaling in Random Networks. Science.

[87] Van Diessen E., Diederen S.J.H., Braun K.P.J., Jansen F.E., Stam C.J. (2013). Functional and structural brain networks in epilepsy: What have we learned?. Epilepsia.

[88] Telesford Q.K., Simpson S.L., Burdette J.H., Hayasaka S., Laurienti P.J. (2011). The Brain as a Complex System: Using Network Science as a Tool for Understanding the Brain. Brain Connect..

[89] Bernhardt C., Bonilha L., Gross D.W. (2015). Network analysis for a network disorder: The emerging role of graph theory in the study of epilepsy. Epilepsy Behav..

[90] Ortega G.J., Sola R.G., Pastor J. (2008). Complex network analysis of human ECoG data. Neurosci. Lett..

[91] Ponten S.C., Bartolomei F., Stam C.J. (2007). Small-world networks and epilepsy: Graph theoretical analysis of intracerebrally recorded mesial temporal lobe seizures. Clin. Neurophysiol..

[92] Wilke C., Worrell G., He B. (2011). Graph analysis of epileptogenic networks in human partial epilepsy: Graph Analysis of Epileptogenic Networks. Epilepsia.

[93] Douw L., van Dellen E., de Groot M., Heimans J.J., Klein M., Stam C.J., Reijneveld J.C. (2010). Epilepsy is related to theta band brain connectivity and network topology in brain tumor patients. BMC Neurosci..

[94] Van Dellen E., Douw L., Baayen J.C., Heimans J.J., Ponten S.C., Vandertop W.P., Velis D.N., Stam C.J., Reijneveld J.C. (2009). Long-Term Effects of Temporal Lobe Epilepsy on Local Neural Networks: A Graph Theoretical Analysis of Corticography Recordings. PLoS ONE.

[95] Yasuda C.L., Chen Z., Beltramini G.C., Coan A.C., Morita M.E., Kubota B., Bergo F., Beaulieu C., Cendes F., Gross D.W. (2015). Aberrant topological patterns of brain structural network in temporal lobe epilepsy. Epilepsia.

[96] Zhang J., Small M. (2006). Complex Network from Pseudoperiodic Time Series: Topology versus Dynamics. Phys. Rev. Lett..

[97] Lacasa L., Luque B., Ballesteros F., Luque J., Nuno J.C. (2008). From time series to complex networks: The visibility graph. Proc. Natl. Acad. Sci. USA.

[98] Zhu G., Li Y., Wen P.P. (2014). Epileptic seizure detection in EEGs signals using a fast weighted horizontal visibility algorithm. Comput. Methods Programs Biomed..

[99] Wang B., Meng L. (2016). Functional brain network alterations in epilepsy: A magnetoencephalography study. Epilepsy Res..

[100] Diykh M., Li Y., Wen P. (2017). Classify epileptic EEG signals using weighted complex networks based community structure detection. Expert Syst. Appl..

[101] Wang L., Long X., Arends J.B.A.M., Aarts R.M. (2017). EEG analysis of seizure patterns using visibility graphs for detection of generalized seizures. J. Neurosci. Methods.

[102] Bogaarts J.G., Hilkman D.M.W., Gommer E.D., van Kranen-Mastenbroek V.H.J.M., Reulen J.P.H. (2016). Improved epileptic seizure detection combining dynamic feature normalization with EEG novelty detection. Med. Biol. Eng. Comput..

[103] Mathieson S., Rennie J., Livingstone V., Temko A., Low E., Pressler R.M., Boylan G.B. (2016). In-depth performance analysis of an EEG based neonatal seizure detection algorithm. Clin. Neurophysiol..

[104] Ansari A.H., Cherian P.J., Dereymaeker A., Matic V., Jansen K., De Wispelaere L., Dielman C., Vervisch J., Swarte R.M., Govaert P. (2016). Improved multi-stage neonatal seizure detection using a heuristic classifier and a data-driven post-processor. Clin. Neurophysiol..

[105] Wulsin D.F., Fox E.B., Litt B. (2014). Modeling the complex dynamics and changing correlations of epileptic events. Artif. Intell..

[106] Andrzejak R.G., Lehnertz K., Mormann F., Rieke C., David P., Elger C.E. (2001). Indications of nonlinear deterministic and finite-dimensional structures in time series of brain electrical activity: Dependence on recording region and brain state. Phys. Rev. E.

[107] Shoeb A., Guttag J. Application of Machine Learning to Epileptic Seizure Onset Detection. Presented at the 27th International Conference on Machine Learning (ICML).

[108] Klatt J., Feldwisch-Drentrup H., Ihle M., Navarro V., Neufang M., Teixeira C., Adam C., Valderrama M., Alvarado-Rojas C., Witon A. (2012). The EPILEPSIAE database: An extensive electroencephalography database of epilepsy patients. Epilepsia.

[109] Baldassano S.N., Brinkmann B.H., Ung H., Blevins T., Conrad E.C., Leyde K., Cook M.J., Khambhati A.N., Wagenaar J.B., Worrell G.A. (2017). Crowdsourcing seizure detection: Algorithm development and validation on human implanted device recordings. Brain.

[110] Obeid I., Picone J. (2016). The Temple University Hospital EEG Data Corpus. Front. Neurosci..

[111] Chen H., Xue M., Mei Z., Oetomo S.B., Chen W. (2016). A Review of Wearable Sensor Systems for Monitoring Body Movements of Neonates. Sensors.

[112] Chen H., Gu X., Mei Z., Xu K., Yan K., Lu C., Wang L., Shu F., Xu Q., Oetomo S.B. A wearable sensor system for neonatal seizure monitoring. Presented at the 2017 IEEE 14th International Conference on Wearable and Implantable Body Sensor Networks (BSN).

[113] Coosemans J., Hermans B., Puers R. (2006). Integrating wireless ECG monitoring in textiles. Sens. Actuators Phys..

[114] Yang B., Yu C., Dong Y. (2016). Capacitively Coupled Electrocardiogram Measuring System and Noise Reduction by Singular Spectrum Analysis. IEEE Sens. J..

[115] Jung H.-C., Moon J.H., Baek D.H., Lee J.H., Choi Y.Y., Hong J.S., Lee S.H. (2012). CNT/PDMS Composite Flexible Dry Electrodesfor Long-Term ECG Monitoring. IEEE Trans. Biomed. Eng..

[116] Ren L., Jiang Q., Chen K., Chen Z., Pan C., Jiang L. (2016). Fabrication of a Micro-Needle Array Electrode by Thermal Drawing for Bio-Signals Monitoring. Sensors.

[117] Pang C., Lee C., Suh K.-Y. (2013). Recent advances in flexible sensors for wearable and implantable devices: Review. J. Appl. Polym. Sci..

[118] Yokus M.A., Jur J.S. (2016). Fabric-Based Wearable Dry Electrodes for Body Surface Biopotential Recording. IEEE Trans. Biomed. Eng..

[119] Nijsen T.M., Aarts R.M., Cluitmans P.J., Griep P.A. (2010). Time-Frequency Analysis of Accelerometry Data for Detection of Myoclonic Seizures. IEEE Trans. Inf. Technol. Biomed..

[120] Poh M.-Z., Loddenkemper T., Reinsberger C., Swenson N.C., Goyal S., Sabtala M.C., Madsen J.R., Picard R.W. (2012). Convulsive seizure detection using a wrist-worn electrodermal activity and accelerometry biosensor: Wrist-Worn Convulsive Seizure Detection. Epilepsia.

[121] Szabó C.Á., Morgan L.C., Karkar K.M., Leary L.D., Lie O.V., Girouard M., Cavazos J.E. (2015). Electromyography-based seizure detector: Preliminary results comparing a generalized tonic-clonic seizure detection algorithm to video-EEG recordings. Epilepsia.

[122] Milošević M., Van de Vel A., Bonroy B., Ceulemans B., Lagae L., Vanrumste B., Van Huffel S. (2016). Automated Detection of Tonic–Clonic Seizures Using 3-D Accelerometry and Surface Electromyography in Pediatric Patients. IEEE J. Biomed. Health Inform..

[123] Goldenholz D.M., Kuhn A., Austermuehle A., Bachler M., Mayer C., Wassertheurer S., Inati S.K., Theodore W.H. (2017). Long-term monitoring of cardiorespiratory patterns in drug-resistant epilepsy. Epilepsia.

